# A deletion in the RD105 region confers resistance to multiple drugs in *Mycobacterium tuberculosis*

**DOI:** 10.1186/s12915-019-0628-6

**Published:** 2019-01-25

**Authors:** Lianhua Qin, Jie Wang, Junmei Lu, Hua Yang, Ruijuan Zheng, Zhonghua Liu, Xiaochen Huang, Yonghong Feng, Zhongyi Hu, Baoxue Ge

**Affiliations:** Shanghai Key Laboratory of Tuberculosis, Clinic and Research Center of Tuberculosis, Shanghai Pulmonary Hospital, Tongji University School of Medicine, Shanghai, 200433 China

**Keywords:** *Mycobacterium tuberculosis*, RD105 region, Rv0071/74-9 m fusion gene, Multiple-drug resistance

## Abstract

**Background:**

The emergence of drug-resistant strains of *Mycobacterium tuberculosis* (Mtb), especially those that are multidrug resistant poses a serious threat to global tuberculosis control. However, the mechanism underlying the occurrence of drug resistance against more than one drug is poorly understood. Given that the Beijing/W strains are associated with outbreaks and multidrug resistance, they may harbor a genetic advantage and provide useful insight into the disease. One marker found in all Beijing/W Mtb strains is a deletion of RD105 region that results in a gene fusion, Rv0071/74, with a variable number (3–9 m) of VDP (V: Val, D: Asp; P: Pro) repeats (coded by gtggacccg repeat sequences) at the N-terminal. Here, we report that this variable number of VDP repeats in Rv0071/74 regulates the development of multidrug resistance.

**Results:**

We collected and analyzed 1255 Beijing/W clinical strains. The results showed that the number of VDP repeats in Rv0071/74 was related to the development of multidrug resistance, and the deletion of Rv0071/74-9 m from Beijing/W clinical strain restored drug susceptibility. Rv0071/74-9 m also increased resistance to multiple drugs when transferred to different mycobacterial strains. Cell-free assays indicate that the domain carrying 4–9 VDP repeats (4–9 m) showed a variable binding affinity with peptidoglycan and Rv0071/74 cleaves peptidoglycan. Furthermore, Rv0071/74-9 m increased cell wall thickness and reduced the intracellular concentration of antibiotics.

**Conclusions:**

These findings not only identify Rv0071/74 with VDP repeats as a newly identified multidrug resistance gene but also provide a new model for the development of multiple drug resistance.

**Electronic supplementary material:**

The online version of this article (10.1186/s12915-019-0628-6) contains supplementary material, which is available to authorized users.

## Background

Tuberculosis is a global health concern. Almost one third of individuals worldwide are infected with the pathogen *Mycobacterium tuberculosis* (Mtb) and are at risk of developing tuberculosis disease during their lifetime [1]. The emergence of drug-resistant strains of *Mtb*, especially those that are multidrug resistant (MDR) and extensively drug resistant (XDR), has posed a serious threat to global tuberculosis control. Given the alarming rise of resistance to tuberculosis drugs worldwide, the identification of resistance gene is critical for the future of tuberculosis control. Several gene mutations in specific loci of the *Mtb* genome have been reported as the basis for drug resistance and as drug targets for the development of anti-tuberculosis drugs [2–5]. However, these known resistance genes are mainly for single drug. There is little evidence that single-locus mutations confer resistance to multiple drugs.

**Fig. 1:**
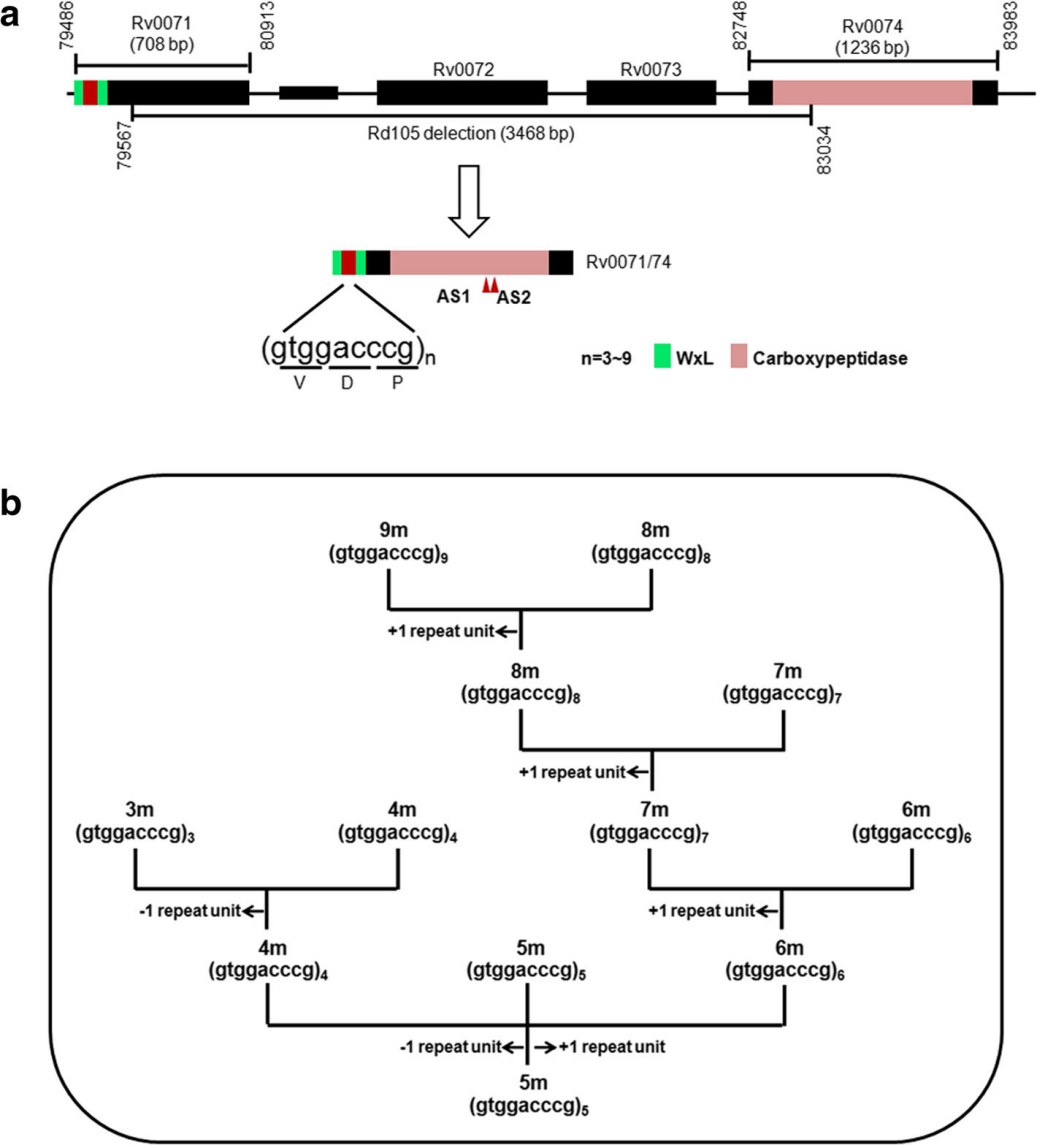
Diagram depicting Rv0071/74-9 m. **a** Organization of the fusion gene Rv0071/74 generated by the deletion of RD105 in Beijing clinical strains. The 1032 bp gene includes the 1–84 bp region from Rv0071 and the 288–1236 bp region from Rv0074. Variable 9-bp sequence repeats (microsatellites) are located at the 5′end of Rv0071/74. **b** Polymorphism distribution of 9-bp sequence repeats site in the tested Beijing clinical strains. Since reference strain H_37_Rv contains 5 of 9-bp sequence repeats, 5 m (motif) is used as an initial point

A genetically related group of *Mtb* strains called Beijing/W is widespread in many regions of the world [6–9]. Strains from this group have been reported to be associated with drug resistance [10, 11]. A deletion of RD105 region is found in all Beijing/W *Mtb* strains and thus serves as a marker of this group [12, 13]. The RD105 region is 3467 bp in size and includes a truncated C-terminal of Rv0071, the full-length Rv0072, full-length Rv0073, and a truncated N-terminal Rv0074 (Fig. 1a). Deletion of the RD105 in Beijing/W *Mtb* strain generates a fusion gene Rv0071/74 that contains a 1–84 bp region of Rv0071 and a 288–1236 bp region of Rv0074 (Additional file 1: Figure S1). The 1–84 bp region of Rv0071 includes variable 9-bp sequence (gtggacccg, coding VDP) repeats plus a WxL domain [14–17] ahead and the 288–1236 bp region of Rv0074, which encodes an amidohydrolase-like family protein [18]. However, the genetic advantage of deletion of the RD105 in Beijing/W strains is unknown. So, we aimed to define the association of RD105 deletion with drug resistance in *Mtb*.

## Results

### Fusion gene Rv0071/74 confers resistance to multiple drugs

**Fig. 2:**
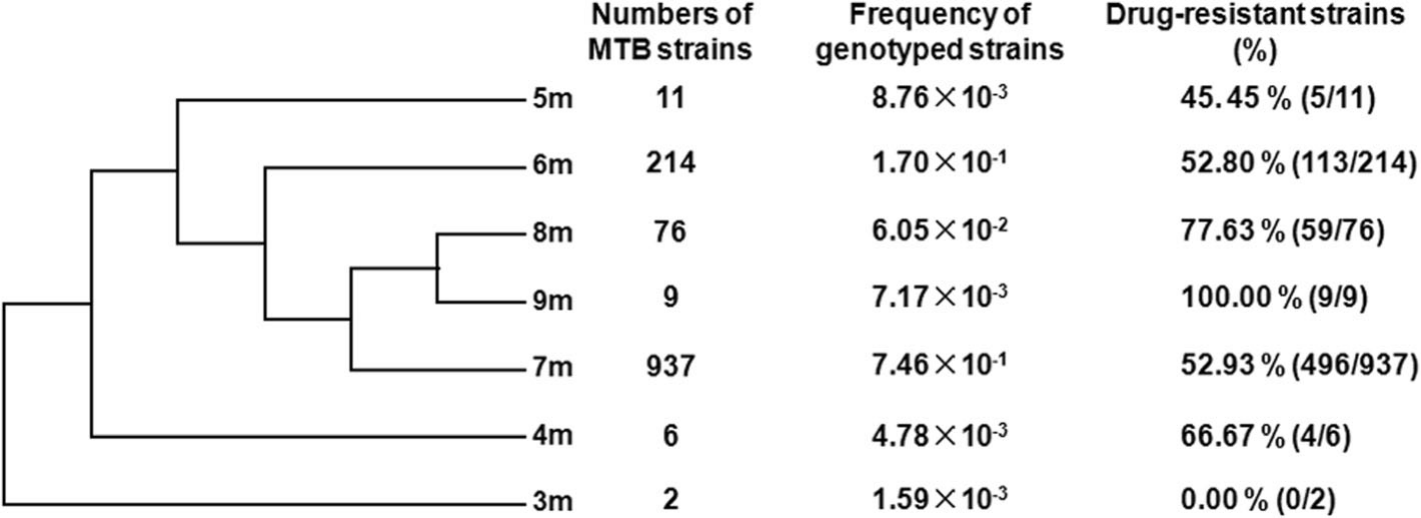
The phylogeny tree of variable 9-bp sequence repeats. The phylogeny tree of variable 9-bp sequence (gtggacccg,) repeats was constructed based upon the aligned sequence using MEGA 6.06 software

We analyzed 1508 *Mtb* clinical strains, from which 1255 of these strains were found to be Beijing/W strains as detected by deletion-targeted multiplex PCR (DTM-PCR) (Additional file 1: Figure S2). In Beijing/W strains, a variable number ranging from 3 to 9 of 9 bp repeats were found at the N-terminal of the Rv0071/74 fusion gene (Fig. 1 and Additional file 1: Figure S1A). Strains carrying 4–9 of 9 bp repeats (4–9 m) showed a different drug resistance, but no detectable drug resistance was observed in the tested Beijing/W clinical strains carrying the 3 m allele (Table 1 and Fig. 2). To understand the correlation between number of tandem repeats and drug-sensitive phenotypes, the phylogeny tree of variable 9-bp sequence (gtggacccg,) repeats was constructed (Fig. 2). Two major groups were clustered according to genotype: 3 m with no drug resistance in the tested strains was divided into a separate group, other 6 alleles (4 m, 5 m, 6 m, 7 m, 8 m, and 9 m) with drug resistance against multiple drugs were grouped together. The closest genetic distance was observed between 8 m (77.63%) and 9 m (100%) with high degree of drug resistance in the tested strains (Fig. 2). Except 4 m allele, the percentage of drug-resistant strains increases as the number of tandem repeats increases. Especially, all the Beijing/W strains carrying the Rv0071/74 fusion gene with 9 of 9 bp repeats (Rv0071/74-9 m) showed a high resistance against multiple drugs, including streptomycin (SM), rifampicin (RFP), isoniazid (isonicotinic acid hydrazide, INH), ethambutol (EMB), ofloxacin (OFX), and amikacin (AMK) (Table 1 and Fig. 2).

**Table 1:** Percentage of drug-resistant Beijing/W clinical strains carrying various 9 bp repeats to different drugs

Drug	Drug resistance strains (%)						
	3 m (*n* = 2)	4 m (*n* = 6)	5 m (*n* = 11)	6 m (*n* = 214)	7 m (*n* = 937)	8 m (*n* = 76)	9 m (*n* = 9)
Streptomycin	0	0	36.36	31.78	40.23	43.42	100
Isoniazid	0	66.67	45.45	38.32	50.16	51.32	100
Rifampicin	0	0	19.41	25.7	38.21	43.13	100
Ethambutol	0	0	18.18	21.5	29.16	27.63	77.78
Ofloxacin	0	16.67	19.13	30.37	34.58	42.11	66.67
Amikacin	0	0	0	8.88	11.74	17.11	63.33

Total: *n* = 1255

To test whether the presence of 9 bp repeats in the 5′ region is linked to the gene expression, we analyzed the mRNA level of Rv0071/74 from Beijing/W clinical strains carrying various 9 bp repeats. Reference strain H_37_Rv (wild Rv0074) was used as a control. No significant difference was found for the transcriptional level of Rv0071/74 (Additional file 1: Figure S3), suggesting that the number of 9 bp repeats is not associated with the expression of Rv0071/74.

We next examined whether the observed multiple drug resistance of Rv0071/74-9 m is caused by known drug-resistance gene mutations. All Beijing/W strains with Rv0071/74-9 m were selected to be tested. Region of drug-resistant gene for SM (rpsL and rrs1), RFP (rpoB), INH (katG and inhA), OFL (gyrA), and AMK (rrs2) were amplified by conventional PCR, and the point mutations of these genes were analyzed by sequencing assay [19, 20]. We found that some classical drug-resistance-related mutations are absent in the Rv0071/74-9 m strains and yet the strains still showed resistance to multiple drugs (Table 2). These results suggest that the single point mutation of known drug resistance genes may not be responsible for multiple drug resistances of Rv0071/74-9 m.

**Table 2:** Mutations of drug-resistant genes in Beijing/W clinical strains carrying 9 m

Strains	SM			RFP		INH			EMB		OFL		AMK	
	MIC (μg/mL)	Gene		MIC (μg/mL)	Gene	MIC (μg/mL)	Gene		MIC (μg/mL)	Gene	MIC (μg/mL)	Gene	MIC (μg/mL)	Gene
		rpsL	rrs1		rpoB		katG	inhA		embB		gyrA		rrs2
1	16	–	–	> 32		1	S315 T AGC/ACC	–	4	–	16	D94G GAC/GGC	0.5	–
2	32	–	A514C	> 32	S531 L TCG/TGG	8	–	–	8	M306I ATG/ATC	32	D94N GAC/AAC	1	–
3	16	–	–	> 32	S531 L TCG/TGG	2	S315 T AGC/ACC	–	4	–	1	–	2	–
4	4	–	C513T	4	D516V GAC/GTC	2	S315 T AGC/ACC	I21V ATC/GTC	1	–	2	D94G GAC/GGC	2	–
5	4	–	C513T	8	D516V GAC/GTC	4	S315 T AGC/ACC	–	0.5	–	2	–	0.5	–
6	> 32	–	A514C	> 32	S531 L TCG/TGG	8	S315 T AGC/ACC	–	4	–	0.25	–	0.5	–
7	4	–	C513T	8	D516V GAC/GTC	4	S315 T AGC/ACC	–	1	–	0.25	–	1	–
8	4	–	C513T	4	D516V GAC/GTC	2	S315 T AGC/ACC	–	0.5	–	0.25	–	2	–
9	16	–	–	> 32	S531 L TCG/TGG	1	S315 T AGC/ACC	C-15 T	4	–	16	D94G GAC/GGC	2	–

*SM* streptomycin, *RFP* rifampicin, *INH* isoniazid, *EMB* ethambutol, *AMK* amikacin, *OFX* ofloxacin

To further determine whether the Rv0071/74-9 m fusion gene confer the antibiotic resistance in mycobacteria, we cloned the Rv0071/74 fusion gene with 3 m or 9 m into plasmid pVV16 and then transformed these constructs into different mycobacterial strains including *M. smegmatis* (ATCC 19420), *M. marinum* (ATCC 927), and *M. tuberculosis* H_37_Ra, which lack the Rv0071/74 fusion gene. No significant difference was observed for the growth rate of these recombinant strains (data not shown), but recombinant mycobacterial strains carrying Rv0071/74-9 m showed markedly increased resistance to multiple drugs (Table 3). In contrast, recombinant strains expressing the Rv0071/74 with 3 m or Rv0071/Rv0074 alone showed no significant gain of drug resistance (Additional file 2: Table S3). Lastly, we generated Rv0071/74-9 m-null mutant by the phage transduction method (Additional file 1: Figure S4A-C). Deletion of Rv0071/74-9 m from Beijing/W strains caused no significant difference for growth rate (data not shown), but markedly reduced resistance to multiple anti-tuberculosis drugs (Table 4). The restoration of Rv0071/74-9 m allele in Rv0071/74-9 m-null mutant (△Rv0071/74-9 m:: Rv0071/74-9 m) restored the mutant to resistance to the tested drugs (Table 4). Together, these results suggest that the Rv0071/74 fusion gene with 9 m (Rv0071/74-9 m) may function as a multidrug resistance gene.

**Table 3:** Drug susceptibility of mycobacterial strains transformed with Rv00171/74-9 m

Strain	MIC (μg/mL)								
	SM	RFP	INH	EMB	OFX	AMK	CFZM	CFXS	LZD
*M. smegmatis* + pVV16	1	16	> 8	≤ 0.25	0.5	1	4	1	0.125
*M. smegmatis +* pVV16::3 m	1	16	> 8	≤ 0.25	0.5	0.5	4	1	0.125
*M. smegmatis +* pVV16::9 m	> 32	> 32	> 8	> 32	4	> 32	> 8	> 64	2
*M. marinum* + pVV16	8	0.5	4	2	4	1	2	8	1
*M. marinum +* pVV16::3 m	8	0.5	4	2	4	1	2	8	1
*M. marinum +* pVV16::9 m	> 32	4	> 8	16	32	4	8	64	4
*H37Ra* + pVV16	4	< 0.25	0.125	0.5	0.5	2	2	8	1
*H37Ra +* pVV16::3 m	4	< 0.25	0.25	0.5	0.5	2	1	16	1
*H37Ra +* pVV16::9 m	32	1	1	4	4	8	8	> 64	4

*SM* streptomycin, *RFP* rifampicin, *INH* isoniazid, *EMB* ethambutol, *OFX* ofloxacin, *AMK* amikacin, *CFZM* ceftazidime, *CFXS* cefoxitin sodium, *LZD* linezolid

**Table 4:** Drug susceptibility of deletion mutant of Beijing/W clinical strains carrying 9 m

Strain	MIC (μg/mL)								
	SM	RFP	INH	EMB	OFX	AMK	CFZM	CFXS	LZD
Wild type	32	> 32	8	8	16	2	4	> 64	> 8
△Rv0071/74-9 m	4	16	4	1	4	0.125	0.5	8	0.25
△Rv0071/74-9 m:: Rv0071/74-9 m	> 32	32	8	16	16	4	8	> 64	> 8

*SM* streptomycin, *RFP* rifampicin, *INH* isoniazid, *EMB* ethambutol, *OFX* ofloxacin, *AMK* amikacin, *CFZM* ceftazidime, *CFXS* cefoxitin sodium, *LZD* linezolid

### Fusion protein Rv0071/74 binds with and cleaves peptidoglycan

**Fig. 3:**
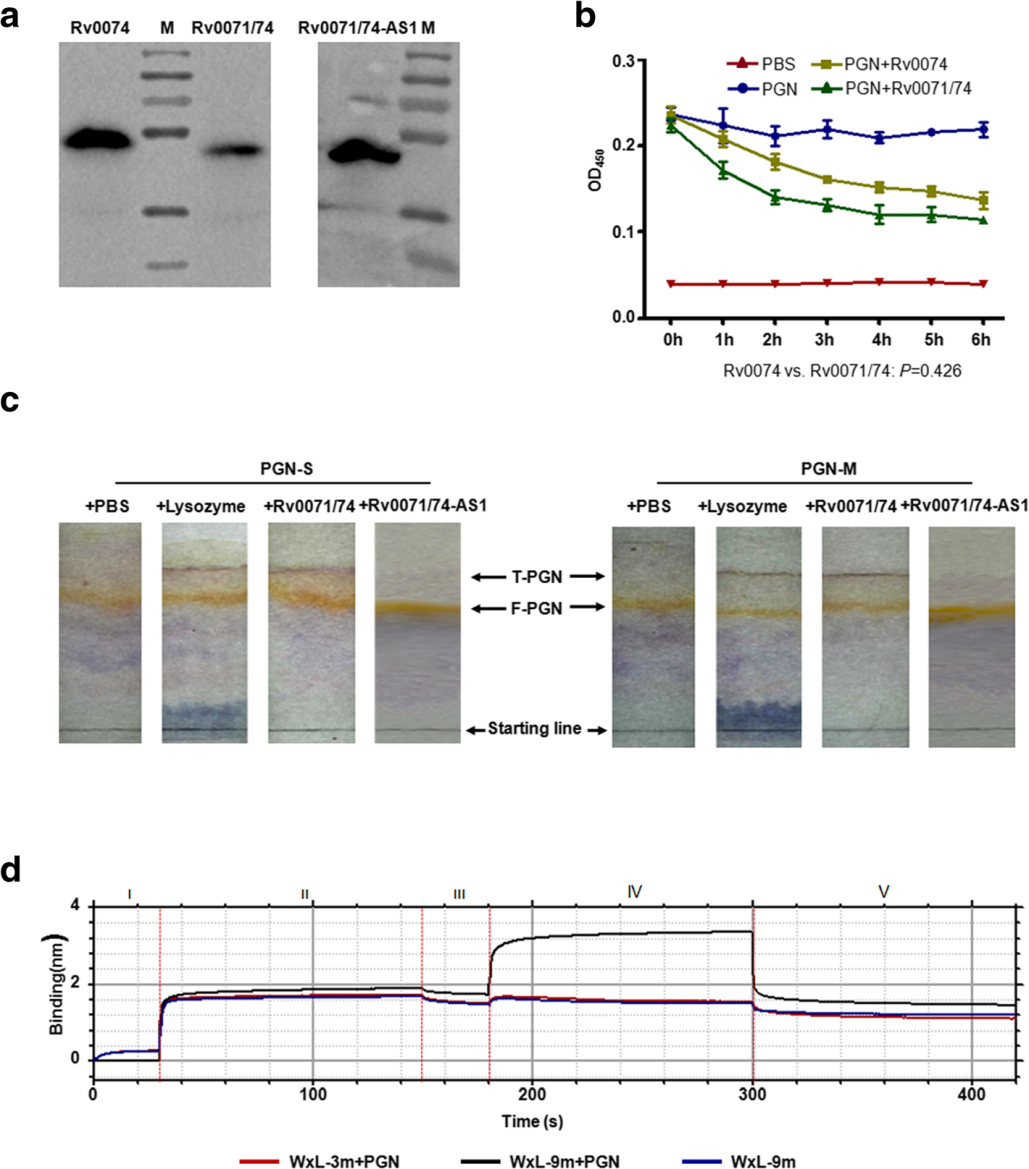
Rv0071/74 binds with and cleaves peptidoglycan (PGN). **a** Purified protein Rv0071/74, Rv0074 and Rv0071/74-AS1 mutant in SDS-PAGE gel stained with Coomassie blue. M: protein ladder. **b**, **c** OD_450_ analysis (Additional file 4: Table S5) (**b**) or liquid chromatography analysis (**c**) of PGN-*S. aureus* (S) or PGN-*M. smegmatis* (M) incubated with purified Rv0071/74 protein. Data shown are representative of three independent experiments. Error bars, means ± SD. F: full-length; T: truncated. **d** Processed kinetic interaction of WxL domain with PGN including baseline (phase I), loading the WxL peptide (phase II), removing unbound peptide (phase III), association of peptide and PGN (phase IV), and dissociation of peptide and PGN (phase V)

Quaternary structure modeling analysis of the Rv0071/74 fusion protein revealed that it has a high homology to a carboxypeptidase from *Caulobacter crescentus* (Additional file 1: Figure S5A). The carboxypeptidase is associated with cell membranes and cleaves the C-terminal d-alanine residue of UDP-muramyl-pentapeptide of peptidoglycan (PGN) in *C*. *crescentus* [21, 22]. Indeed, purified Rv0071/74-9 m protein can directly cleave the PGN isolated from *S*. *aureus* or *M. smegmatis*, as measured by OD_450_ or liquid chromatography (Fig. 3a–c). Furthermore, carboxypeptidase Cc2672 contains two active-site amino acid residues that are located in β-sheet regions of the protein (Additional file 1: Figure S5B). Structure modeling analysis of the Rv0071/74 protein also revealed two conserved active-site amino acid residues (AS1 and AS2) and analogous active regions similar to those of the carboxypeptidase Cc2672 (Additional file 1: Figure S5B). Point mutations in these two active-site amino acid residues all change the modeled quaternary structure of Rv0071/74 (Additional file 1: Figure S5B). We mutated these two active-site amino acid residues individually in Rv0071/74-9 m. The mutant protein was expressed at the same level as the non-mutant protein in *M. smegmatis* (data not shown). The purified mutant protein Rv0071/74 lost the carboxypeptidase (Fig. 3a, c). Recombinant *M. smegmatis* strains carrying these mutated Rv0071/74-9 m alleles lost drug resistance as compared with the strains containing pVV16::9 m (Additional file 2: Table S4).

The Rv0071/74 fusion protein inherits most (including microsatellites) of the WxL domain, which is responsible for the binding to PGN [14–17] (Fig. 1). To evaluate the association of Rv0071/74-9 m with PGN, we synthesized variable-VDP repeats-associated WxL peptides, MSSITVS-(VDP)_n_-VDAVVAVGRARR (*n* = 3–9), and labeled with biotin at the N-terminal. The biotin-labeled peptide was then incubated with PGN from *S. aureus* and analyzed by a ForteBio’s Octet platform. The WxL domain with 9 VDP repeats showed much higher affinity with PGN as compared to WxL-3 m (Fig. 3d). In addition, WxL domain carrying 4–8 VDP repeats (4-8 m) showed a variable binding affinity with PGN (Additional file 1: Figure S6), which may explain the variable degree of multiple drug resistance observed in strains carrying 4–8 of 9 bp repeats (4–8 m).

### Rv0071/74-9 m increases cell wall thickness and reduces intracellular drug concentration

**Fig. 4:**
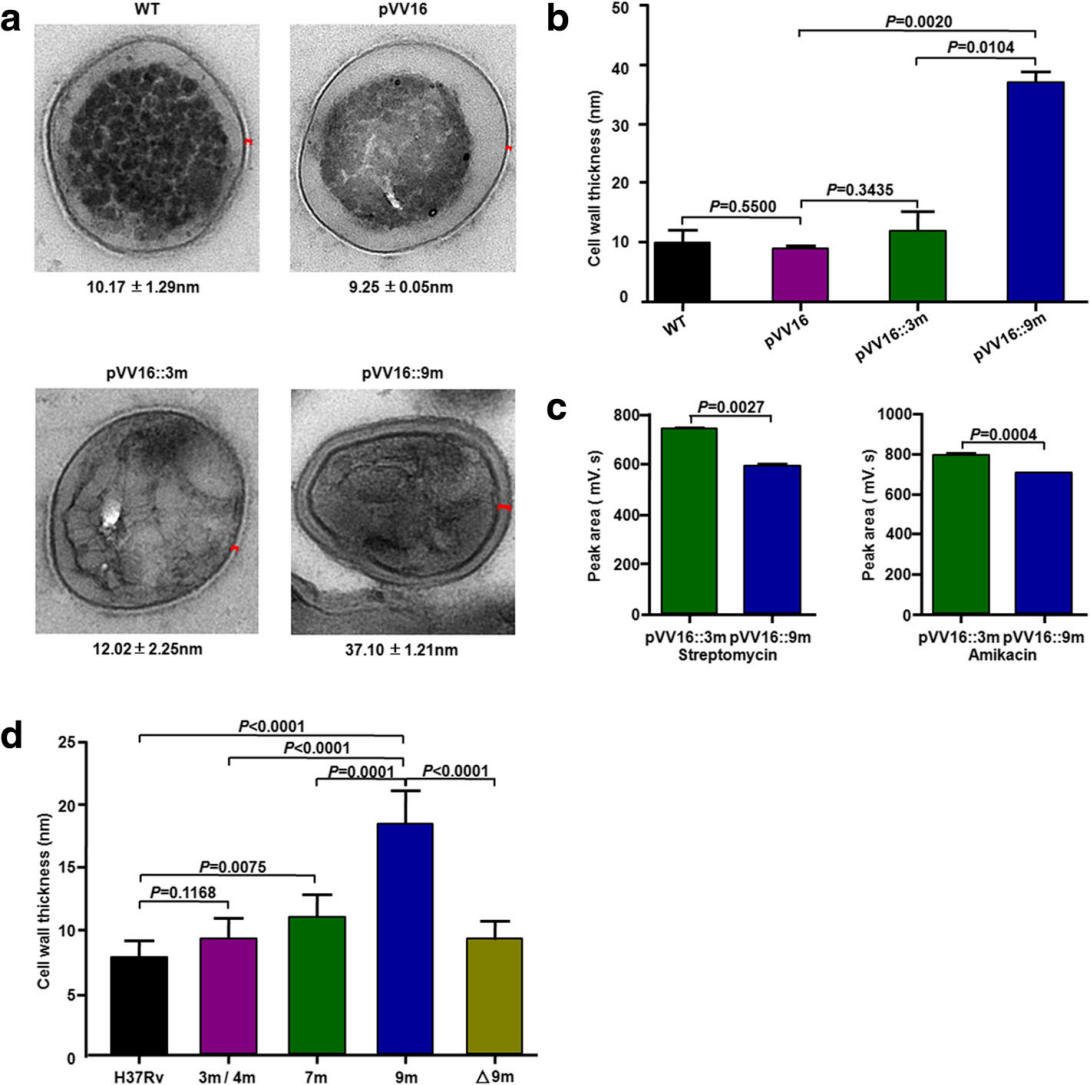
WxL-9 m increases cell wall thickness and reduces intracellular drug concentration. **a** Ultrastructural morphology of different recombinant *M. smegmatis* strains analyzed by transmission electron microscopy (TEM). Five cells that had been cut were chosen for each strain, and cell-wall thickness was determined at four sites for each cell. The cell wall thickness of recombined strains is shown as the means ± SD in nanometers. **b** Statistic analysis of cell wall thickness of different recombinant *M. smegmatis* strains as in **a**. **c** Mass spectrometry analysis of intracellular drug concentration of streptomycin or amikacin in *M. smegmatis* transformed with pVV16::9 m or pVV16::3 m. Data shown are representative of three independent experiments. Error bars, means ± SD. **d** Statistic analysis of cell wall thickness of different *Mtb* strains analyzed by TEM: H_37_Rv (wild-type Rv0074) and Beijing/W *Mtb* with 3 m/4 m (drug-sensitive strains), 7 m (MDR strains), 9 m, and △9 m (△Rv0071/74-9 m). Five cells that had been cut were chosen for each strain, and cell-wall thickness was determined at four sites for each cell. Error bars, means ± SD

Transmission electron microscopy (TEM) was used to evaluate the differences between *M. smegmatis* containing pVV16::9 m and control groups containing wild-type *M. smegmatis* and *M. smegmatis* with only the pVV16 plasmid or pVV16::3 m. Under TEM, marked differences were observed in the thickness of cell walls: 37.10 ± 1.21 nm for *M. smegmatis* containing pVV16::9 m, but < 15 nm for the control groups (Fig. 4a, b).

To examine whether the increased thickness of the cell wall in *M. smegmatis*::Rv0071/74-9 m affects the permeability of drugs, the intracellular concentration of streptomycin or amikacin in recombinant *M. smegmatis*::Rv0071/74-9 m was analyzed by mass spectrometry. *M. smegmatis* transformed with pVV16::9 m had a much lower intracellular drug concentration of streptomycin and amikacin than the control *M smegmatis* carrying the recombinant pVV16::3 m plasmid (Fig. 4c).

To evaluate the differences between Beijing/W *Mtb* with 9 m and control groups containing H_37_Rv (wild-type Rv0074) and Beijing/W *Mtb* with Rv0071/74-3 m or 7 m, cell wall thickness of these different *Mtb* strains were also analyzed by TEM. The cell walls of Beijing/W *Mtb* strains with 9 m are much thicker than those of control groups under TEM (*p* < 0.0001) (Fig. 4d). Deletion of Rv0071/74-9 m from Beijing/W strains significantly decreased the cell wall thickness (*p* < 0.0001) (Fig. 4d) and then markedly reduced resistance to multiple anti-tuberculosis drugs (Table 4).

## Discussion

Multidrug-resistant *Mtb* poses the greatest obstacle to the tuberculosis control. A better knowledge of the mechanisms of drug resistance in *Mtb* and the molecular mechanisms involved will stimulate the exploration of new targets for drug activity and drug development. So far, spontaneous point mutations in the specific gene have been characterized as the main basis for *Mtb* drug resistance, especially to RIF and INH [2, 5]. More than 95% RIF resistance is mainly caused by mutations of 81 bp RRDR region (resistance-determining region) of rpoB gene [3, 5]. And the S315 T mutation in the katG gene is the predominant mechanism of INH resistance in *Mtb* [3, 5]*.* Our data also showed this phenomenon (Table 2). The resistance to EMB, SM, OFL, and AMK are primarily found in mutations in the embB, rpsl, gyrA, and rrs genes, respectively. In summary, mutations in these genes are associated with drug resistance in roughly 70–85% of drug resistance isolates of *Mtb* [3, 5]. At present, new genetic mutations of drug resistance have been identified by structural analysis of *Mtb* pan-genome [23]. However, these new resistance genes still require more detailed experimental studies to test their association with resistant phenotypes.

Although point mutations in resistant genes play a major role in *Mtb* drug resistance, it has been reported that other drug-resistant mechanisms such as drug efflux pumps, changes in cell wall permeability, etc. also play an important role in drug tolerance. The development of drug resistance in mycobacteria is complex and controlled by a variety of mechanisms. Multiple mechanisms of resistance are acting in unison in the emergence of drug resistance. Our study here showed a newly identified multidrug resistance gene Rv0071/74, which is generated from the deletion of the RD105 region in Beijing/W strains.

We have found that the Beijing/W strains carrying 3–9 of VDP repeats (4–9 m) showed a different degree of multiple drug resistance. As the number of repeats increases, the percentage of resistant strains also increases. Based on the quaternary structure modeling of the Rv0071/74 fusion protein, Rv0071/74 (101-214aa) has a high homology to a carboxypeptidase Cc2672 from *Caulobacter crescentus* Cb15 (PDB ID: 3MTW) Ad3, 152–269 aa). Furthermore, similar to carboxypeptidase Cc2672, Rv0071/74 protein also revealed two conserved active-site amino acid residues (AS1 and AS2) that are located in β-sheet regions of the protein. Purified recombinant Rv0071/74-9 m or wild-type Rv0074 from non-Beijing strains all can directly cleave the peptidoglycan (PGN) isolated from *S. aureus* or *M. smegmati* in vitro*,* suggesting there is no difference in the carboxypeptidase activity of these proteins (*P* = 0.426). However, compared with wild-type Rv0074 from non-Beijing strain, fusion Rv0071/74 gene generated from the deletion of the RD105 region in Beijing/W strain carries a WxL domain plus a variable number of VDP repeats. WxL domain is known for its ability to interact with PGN [14–17]. Indeed, WxL domain carrying 4–9 VDP repeats (4–9 m) showed a variable binding affinity with PGN. Thus, the variable degree of multiple drug resistance observed in strains carrying variable number of 9 VDP repeats (3–9 m) could be due to a variable binding affinity WxL domain carrying 3–9 VDP repeats with PGN in vivo.

PGN, an essential component of the bacterial cell envelope, provides structural integrity to mycobacterial cells [24, 25]. Since Rv0071/74-9 m can bind and cleave the PGN, we have evaluated its effect on the morphology characteristics of mycobacteria at the ultrastructural level. Our data showed that the thickness of cell walls of Rv0071/74-9 m strains is markedly increased as compared to those of the control group. Thickening of the bacterial cell wall generally increases drug resistance by reducing drug permeability [26–29]. Our results also demonstrated that those mycobacterial strains transformed with pVV16::9 m have a much thicker cell wall, and much lower intracellular drug concentration than the control. The previous studies have revealed that the cell walls in resistant (MDR and XDR) strains are much thicker than those of susceptible strains of *Mtb* under TEM [30, 31]. Moreover, the basal layer of the PG in XDR strains appeared more electron dense and was almost diffused into the intermediate layer, forming the dense thick layer around the plasma membrane [31]. This phenomenon was also observed in *M. smegmatis* containing pVV16::9 m (Fig. 4a) and Beijing/W Mtb with 9 m under TEM.

Thus, the resistance of Rv0071/74-9 m or to multiple drugs could be due to the reduced permeability of the cell wall to multiple drugs. However, the exact mechanism of how the carboxypeptidase activity of the Rv0071/74 fusion protein change antibiotic permeability is still await for further investigation, one possible explanation is that Rv0071/74-9 m fusion protein may bind to PGN through its WxL domain and cleave PGN through its carboxypeptidase activity. This event may enhance the cross-linking of PGN to increase the thickness of the cell wall in mycobacteria, thus reducing the permeability of the cell wall to multiple drugs.

## Conclusions

Tuberculosis remains one of the leading causes of death worldwide. The emergence of drug-resistant strains of *Mtb*, especially those that are multidrug resistant and extensively drug resistant, has posed a serious threat to global tuberculosis control. Given the alarming rise of resistance to tuberculosis drugs worldwide, the identification of resistance gene is critical for the future of tuberculosis control. However, these known resistance genes are mainly for single drug and there is little evidence that single-locus mutations confer resistance to multiple drugs. Our data suggest that a fused Rv0071/74 gene with 9 of 9-bp repeats (Rv0071/74-9 m) increased resistance to multiple drugs by increasing cell wall thickness and reducing the intracellular concentration of antibiotics. Our findings not only reveal a mechanism for the development of multi-drug resistance but also may provide the basis for the design of more effective anti-tuberculosis drugs.

## Methods

### Strain collection and preparation

A total of 1508 *M*. *tuberculosis* strains simultaneously identified by growth characteristics, colony morphology, and growth in the presence of PNB (pnitrobenzoic acid) and TCH (thiophene-2-carboxylic acid hydrazide), and 16SrRNA sequencing [32] were mainly collected mainly from regions of Eastern China, including Shanghai, Jiangsu, Zhejiang, Shandong, Fujian, Anhui, and Jiangxi, by Laboratory of Tuberculosis, Shanghai Pulmonary Hospital. All strains were grown in Sauton culture medium supplemented with 0.5 g/L KH_2_PO_4_, 0.5 g/L MgSO_4_·7H_2_O, 2 g/L citric acid, 0.05 g/L ferric ammonium citrate, 4.0 g/L L-asparagine, 6% glycerol and 0.02% Tween 80. Strains were sterilized at 80 °C for 30 min, and collected by centrifugation (12,000*g* for 5 min). The bacterial pellet was washed three times with sterilized saline and re-collected by centrifugation (12,000*g* for 10 min each time). The ethics approvals were obtained for this study from Shanghai Pulmonary Hospital Ethics Committee (the permit numbers: 2011-fk-03).

### Extraction of bacteria DNA

The total bacterial pellet were resuspended in 50 mL DNA lysis buffer including 10 mmol/L NaCl, 1 mg/mL SDS, 15% Chelex-100, and 1% Tween 20. The mixture was incubated at 50 °C for 1 h, followed by 100 °C for 10 min, then centrifuged (5000*g* for 10 min) to obtain the aqueous phase containing genomic DNA.

### Identification of Beijing strains and microsatellite typing

Beijing strains were identified by deletion-targeted multiplex PCR (DTM-PCR) to detect the genomic deletion RD105, which defines the Beijing family as a separate lineage within *Mtb*. The DTM-PCR primers BP1 (5′-GGAGTCGTTGAGGGTGTTCATCAGCTCAGTC-3′) and BP2 (5′-CGCCAAGGCCGCATAGTCACGGTCG-3′) were used to amplify a 1466 bp product from the non-W-Beijing strains, while BP1 and BP3 (5′-GGTTGCCCACTGGTCGATATGGTGGACTT-3′) were used to amplify a 761-bp fragment from the Beijing genotype [33, 34]. PCR products were separated on 0.8% agarose gels. To detect polymorphisms of the 9-bp sequence repeats locus within Beijing strains, PCR products from Beijing strains were also sequenced on an ABI 3730xl DNA Analyzer according to the procedures by manufacturers (Sangon, Shanghai, China).

### Drug susceptibility test in vitro

The drug susceptibility test of all Beijing strains against the four first-line drugs (SM, INH, RFP, EMB) were carried out by the BACTEC 960 method [35, 36]. The MIC (minimum inhibitory concentration) determination of selected strains was tested in 96-well culture plates containing Middlebrook 7H9 liquid medium supplemented with 0.25% glycerol, 10% oleic acid–albumin-dextrose-catalase, and 0.05% Tween-80, as described by Kumar et al. [37] .The final concentrations of drugs are provided (Additional file 2: Table S1).

### Mutations of drug-resistant genes

Mutation of drug-resistant genes was tested as described previously described [19, 20]. Region of drug-resistant gene for SM (rpsL and rrs1), RFP (rpoB), INH (katG and inhA), OFL (gyrA), and AMK (rrs2) were amplified by conventional PCR. PCR products were sequenced (Sangon, Shanghai, China). Point mutations of drug-resistant genes were analyzed by DNAstar soft. The region and mutations of drug-resistant gene are provided (Additional file 3: Table S2).

### Plasmid construction and bacteria transformation

The Rv0071/74 fusion genes with different alleles were cloned into plasmid pVV16 [38] and then transformed into *M. smegmatis* (ATCC 19420), *M. marinum* (ATCC927), and H37Ra*.* The Rv0071/74-9 m were amplified and cloned into the pET28a expressing plasmid and then transformed into *E*. *coli* BL21 (DE3) strains.

### Morphological observation

*M. smegmatis* and selected recombinant strains were cultured in 96 U well culture plates containing Middlebrook 7H9 liquid medium or on Middlebrook 7H10 agar medium with hygromycin or without hygromycin at 37 °C and were grown until the formation of colonies (about 3–5 days for liquid culture, and 5–7 days for solid culture). Morphological characteristics of tested strains were observed by × 2 magnifier for liquid culture and by an inverted light microscope for solid culture. For ultrastructural characteristics, strains at mid-log phase were collected and analyzed by Tecnai transmission electron microscopy (TEM) with 160 kV according the procedures by manufactures (GOODBIO, Wuhan, China). To analyze ultrastructural morphology of *Mtb* strains with different alleles, 15 *Mtb* strains including H_37_Rv (wild Rv0074) and Beijing/W *Mtb* with 9 m (*n* = 5), 7 m (*n* = 5, MDR strains), 3 m/4 m (*n* = 4, drug-sensitive strains), and △Rv0071/74-9 m strains were also examined by TEM observation. These tested strains were all grown in Middlebrook 7H9 Broth (Difco) supplemented with 0.2% glycerol and 10% Middlebrook OADC Enrichment (Difco), and then harvested in the exponential growth phase. 10^7^–10^8^ bacterial suspension was used for TEM examination. Two-tailed unpaired *t* tests were used to analyze the difference of cell wall thickness between recombinant *M. smegmatis* strains or *Mtb* strains. Statistical significance was defined as *P* < 0.05.

### Generation of Rv0071/74-9 m mutants

The Rv0071/74-9 m-null mutant was generated by the phage transduction method [39]. To construct a transducing phage for *Mtb* Rv0071/74-9 m knockout, the left homolog arm was PCR amplification using primers KOP1 and KOP2. The right homolog arm was PCR amplification using primers KOP3 and KOP4. The PCR products were ligated into *Afl*II/*Xba*I and *Hind*III/*Bgl*II sites of pYUB854. The recombinant transducing phage was used to construct the Rv0071/74-9 m-null MTB as described. The mutant was verified by PCR(P1 and P2) and reverse transcription-PCR (P3 and P4). The Rv0071/74-9 m fusion gene was cloned into plasmid pVV16 [38] and then transformed into the Rv0071/74-9 m-null mutant*.*

### RNA extraction and quantitative real-time PCR

MTB strains (OD600 0.25) at mid-log phase were collected. Total RNA was extracted with TRIzol-Reagent (Invitrogen). Briefly, cell pellet was resuspended in 1 ml Trizol reagent, mixed with 400 ml 0.1 mm Zirconia Beads (Sigma Products) and lysed in a mini-bead beater (Biospec) for three cycles (40 s at maximal speed) with cooling on ice for 1 min between pulses. RNA was extracted according to the protocol of TRIzol-Reagent. The extracted RNA was further followed by DNase I treatment to eliminate DNA contamination. cDNA was synthesized using the PrimerScript II 1st strand cDNA synthesis kit (TaKaRa). Target gene transcript levels were measured by real-time PCR using SYBRH Premix Ex Taq GC (TaKaRa) on applied biosystems 7500 real-time PCR: 95 °C 60 s, 40 cycles of 95 °C 5 s, 62 °C 8 s and 72 °C 20 s, followed by melting curve analysis. The relative transcriptional level was determined by the 2^−ΔΔCt^ method. Reference strain H_37_Rv (wild-type Rv0074) was as a control. The reference gene was 16S rRNA.

### Protein structure analysis

Quaternary structure modeling of Rv0071/74-9 m fusion protein was analyzed online by SWISS-MODEL at http://swissmodel.expasy.org/ [40]. Active regions and active sites were analyzed online in Conserved Domain Database of NCBI (http://www.ncbi.nlm.nih.gov/).

### Binding analysis of WxL domain with peptidoglycan

The binding between WxL domain and peptidoglycan (PGN) was measured using interferometry by a ForteBio’s Octet platform. The WxL daomains from Rv0071/74 were synthesized as peptides with the following sequence MSSITVS-(VDP)_n_-VDAVVAVGRARR (*n* = 3~9), and biotin-labeled at the N terminal. Streptavidin-labeled biosensor was firstly equilibrated incubated in PBS buffer for 30 s, and then loaded with the biotin-labeled peptide (0.5 g/L) in the PBS buffer for 3 min. After the baseline step in PBS buffer for 30 s, the biosensors with the biotin-labeled peptide was placed in the PGN from *S. aureus* (Sigma-Aldrich) in PBS buffer (1 g/ L) for 5 min for association, and then placed in the PBS buffer for 5 min for dissociation. The binding of WxL region to PGN was analyzed with subtracted data (obtained by subtracting background data from raw data) using the Octet data analysis software.

### Peptidoglycan cleavage assay

Protein was purified to nearly SDS-PAGE homogeneity. Protein concentration was determined by Coomassie brilliant blue G-250 method. The purified Rv0071/74-9 m protein (10 μg/well) was incubated with PGN from *S. aureus* at 37 °C for 0, 1, 2, 3, 4, 5, and 6 h. OD450 of each time point was tested at a spectrophotometer (BIOTEK uQuant MQX200). The change of OD450 of each time point was analyzed by GraphPad Prism 5.0 statistical software package (http://www.graphpad.com/prism/prism.htm). For liquid chromatography analysis of PGN cleavage, the PGN-S (from *S. aureus*) or PGN-M (from *M. smegmatis*) was first incubated with the Rv0071/74 protein or lysozyme, a known glycanohydrolase catalyzing hydrolysis of PGN, as a positive control in our study, at 37 °C for 2 h, the samples were loaded on the filter paper and spread for 1 h, and then stained by 5% Ninhydrin.

### Polyclonal antibody preparation and Western blot

Rv0071/74-9 m fusion protein was purified as previously described and used to immunize rabbits for the production of anti-Rv0071/74-9 m fusion protein polyclonal antibody. Standard western blot procedures were used. Cell extracts (50 μg) were separated by 12% SDS–PAGE and then transferred to a polyvinylidene fluoride membrane. The concentration of the primary antibodies used in the corresponding blot were anti-Rv0071/74-9 m (1:1000). Horseradish peroxidase-conjugated goat anti-rabbit polyclonal antibody was used as the secondary antibody at a 1:5000 dilution.

### Growth rate

*M. smegmatis* or recombinant *M. smegmatis* at mid-log phase were diluted to a calculated starting OD600 of 0.25 and added into 96 well culture plates containing Middlebrook 7H9 liquid medium. OD600 of each time point of each strain was tested in real time by Bioscreen C microplate incubator (FP1100-C, Labsystems, USA) at 37 °C for 7 days. Data shown are representative of three duplicates. Error bars, means ± SD. Two-tailed unpaired t-tests were used to analyze the difference of growth rate between recombinant *M. smegmatis* strains. Statistical significance was defined as *P* < 0.05.

### Analysis of intracellular concentrations of drugs

*M. smegmatis* or recombinant *M. smegmatis* at mid-log phase were diluted to a calculated starting OD600 of 0.25 and grown with 1/4 MIC drugs for 48 h at 37 °C. Strains were collected and washed three times with sterilized ddH_2_O. Bacterial pellets were resuspended in 200 μL ddH_2_O. The supernatant containing intracellular drugs was prepared via ultrasonication and loaded on a weak cation-exchange (WCX) solid-phase extraction column. The hydrophilic interaction chromatography (HILIC) was used to retain the drugs on the column for the separation of the analytes. The drugs were detected and quantified with triple quadrupole tandem mass spectrometry with ESI source in the positive ion mode. Quantitation was performed using multiple reaction monitoring (MRM) of the transitions with isepamycin as the internal standard. Data shown are representative of three independent experiments. Error bars, means ± SD. Two-tailed unpaired t-tests were used to analyze the difference of intracellular concentration of drugs between recombinant *M. smegmatis* strains. Statistical significance was defined as *P* < 0.05.

## Additional files

Additional file 1: **Figure S1.** Sequences of fusion protein Rv0071/74 and fusion protein Rv0071/74-9 m in *mycobacterial* analyzed by West-blot. **Figure S2.** Distribution of drug-resistant strains in tested *Mtb* clinical strains. **Figure S3.** RT-PCR analysis of Rv0071/74 fusion gene mRNA expression from different Beijing/W clinical strains. **Figure S4.** Generation of Rv0071/74-9 m mutant. **Figure S5.** Structure modeling of Rv0071/74. Figure S6. Variable Binding of WxL domain with different alleles with PGN. (PDF 716 kb)

Additional file 2: **Table S1.** Final concentrations of antibiotics in drug susceptibility test. **Table S3.** Drug susceptibility of recombinant *M. smegmatis* transformed with Rv0071 or Rv0074. **Table S4.** Drug susceptibility of recombinant *M. smegmatis* transformed with Rv0071/74-9 m carrying point mutation of AS1 or AS2. (DOCX 23 kb)

Additional file 3: **Table S2.** The region and mutations of drug-resistant gene. (XLS 32 kb)

Additional file 4: **Table S5.** The raw values for Fig. 3b. (XLS 25 kb)

## Abbreviations

AMK: Amikacin
DTM-PCR: Deletion-targeted multiplex PCR
EMB: Ethambutol
HILIC: Hydrophilic interaction chromatography
INH: Isoniazid
MDR: Multidrug resistant
MIC: Minimum inhibitory concentration
MRM: Multiple reaction monitoring
Mtb: *Mycobacterium tuberculosis*
OFX: Ofloxacin
PBS: Phosphate-buffered solution
PGN: Peptidoglycan
PNB: Pnitrobenzoic acid
RFP: Rifampicin
SDS-PAGE: Sodium dodecyl sulfate polyacrylamide gel electrophoresis
SM: Streptomycin
TCH: Thiophene-2-carboxylic acid hydrazide
TEM: Transmission electron microscopy
WCX: Weak cation-exchange
XDR: Extensively drug resistant
