# Supplementary material for: A deletion in the RD105 region confers resistance to multiple drugs in Mycobacterium tuberculosis
Source: BMC Biol. 2019 Jan 25;17:7. doi: 10.1186/s12915-019-0628-6 (PMC6347829; doi:10.1186/s12915-019-0628-6)
Supplement: Supplementary file 1 — Figure S1. Sequences of fusion protein Rv0071/74 and fusion protein Rv0071/74-9 m in mycobacterial analyzed by West-blot. Figure S2. Distribution of drug-resistant strains in tested Mtb clinical strains. Figure S3. RT-PCR analysis of Rv0071/74 fusion gene mRNA expression from different Beijing/W clinical strains. Figure S4. Generation of Rv0071/74-9 m mutant. Figure S5. Structure modeling of Rv0071/74. Figure S6. Variable Binding of WxL domain with different alleles with PGN. (PDF 716 kb) [file 12915_2019_628_MOESM1_ESM.pdf]

a

Rv0071	MSSITVSVDPVDPVDPVDPVDPVDAVVA
**Rv0071/74**	MSSITVSVDPVDPVDPVDPVDPVDAVVAVGRARRHAAAALRSGITTIRDLGD
RV0074	VGRARRHAAAALRSGITTIRDLGD
**Rv0071/74**	RDYAALALREEYRQKTTVGPELVVSGPPLTRSGGHCWFLGGVADSVEELVD
RV0074	RDYAALALREEYRQKTTVGPELVVSGPPLTRSGGHCWFLGGVADSVEELVD
**Rv0071/74**	AVQERAARGADWIKVMATGGFVTTASDPWQPQYGSGQLAAVVAAAEQVG
RV0074	AVQERAARGADWIKVMATGGFVTTASDPWQPQYGSGQLAAVVAAAEQVG
**Rv0071/74**	LPVTAHAHATAGIAAAVAAGVDGIEHCTFLSEGSAAASPDVVEAIVAQGVW
RV0074	LPVTAHAHATAGIAAAVAAGVDGIEHCTFLSEGSAAASPDVVEAIVAQGVW
**Rv0071/74**	CGMTIPRVYPEMPENLVAVVQDGWRNIRRLIDAGARVALSTDAGVAPGRRH
RV0074	CGMTIPRVYPEMPENLVAVVQDGWRNIRRLIDAGARVALSTDAGVAPGRRH
**Rv0071/74**	DVLPDDLVYLSRHGFTSTEVLTGATAAAAASCGLGHRKGRIAPGYDADLLA
RV0074	DVLPDDLVYLSRHGFTSTEVLTGATAAAAASCGLGHRKGRIAPGYDADLLA
**Rv0071/74**	VAAGVDHDPAGLCDVKAVWRSGTQVPLQASAVGYNTPS
RV0074	VAAGVDHDPAGLCDVKAVWRSGTQVPLQASAVGYNTPS

b

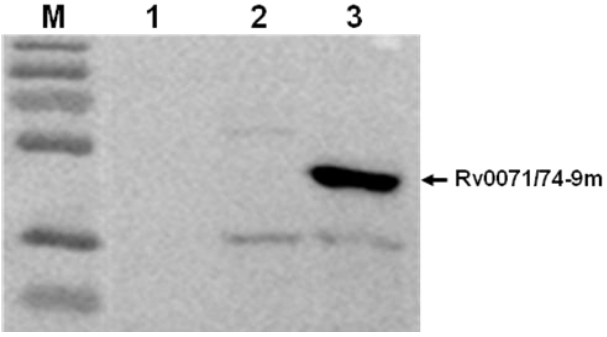

Supplementary Fig. 1 (a) Sequences of fusion protein Rv0071/74. 28 aa from Rv0071 (1-28); 315 aa from Rv0074 (29-343). (b) Fusion protein Rv0071/74-9m in *mycobacterial* analyzed by West-blot. M: protein ladder, 1: *M. smegmatis* (ATCC 19420) , 2: *Mtb* $H_{37}Rv$ 3: Beijing/W *Mtb* strain.

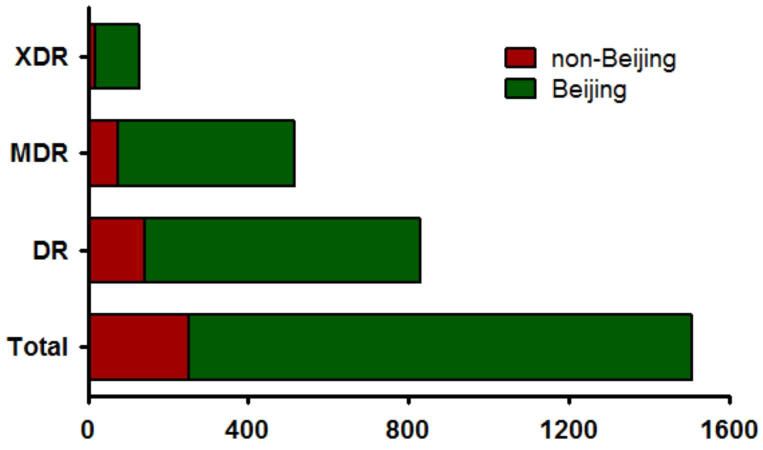

Supplementary Fig. S2 Distribution of drug-resistant strains (drug resistant (DR), multidrug resistant (MDR) and extensively drug resistant (XDR)) in 1508 *Mtb* clinical strains (including 1255 Beijing/W strain).

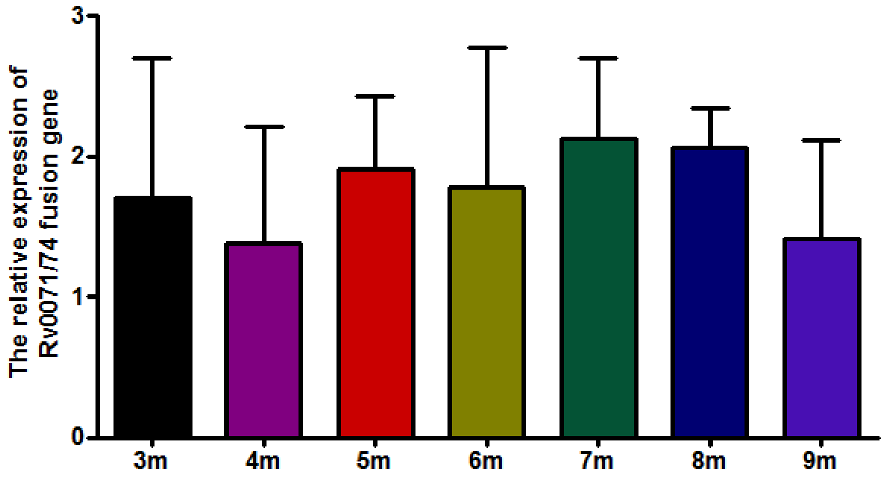

Supplementary Fig. S3 RT-PCR analysis of Rv0071/74 fusion gene mRNA expression from different Beijing/W clinical strains with 3m (n=2) , 4m (n=6), 5m (n=11), 6m (n=11), 7m (n=11), 8m (n=11), and 9m (n=9). Data are from one experiment with 3 biological duplicates. Reference strain $H_{37}Rv$ (wild-type Rv0074) was as a control. The reference gene was 16S rRNA. Error bars, means ± SD.

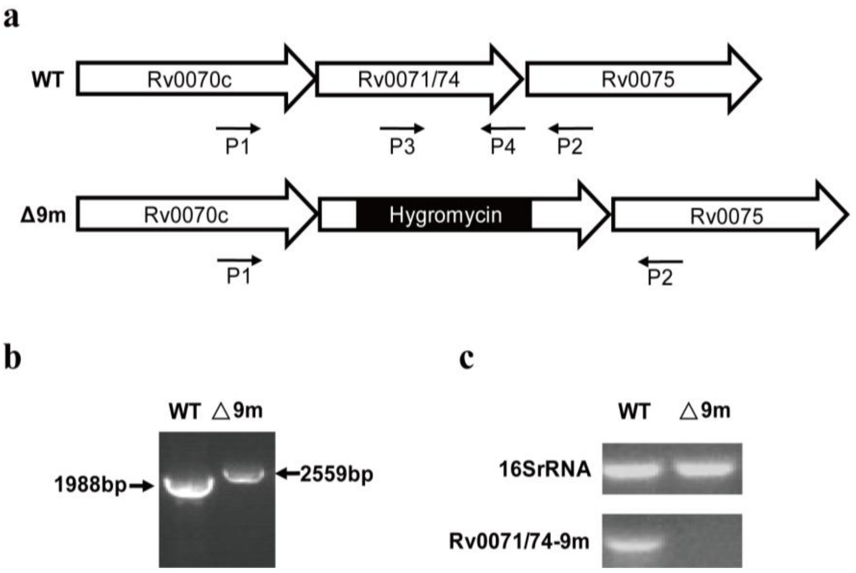

Supplementary Fig. S4 Generation of Rv0071/74-9m mutant (**a**) Schematic diagrams of wild-type (WT) and the Rv0071/74-9m-null (△ Rv0071/74-9m or △ 9m) loci. The mutant was verified by PCR (P1 and P2) and reverse transcription-PCR (P3 and P4). The primers used for PCR are shown as arrows. (**b**) Agarose gel electrophoresis analysis of PCR amplification product of WT strain or △ 9m using primers P1 and P2. (**c**) mRNA from WT strain or △ 9m were reverse transcribed, followed by PCR amplification using primers P3 and P4, and then subjected to agarose gel electrophoresis analysis.

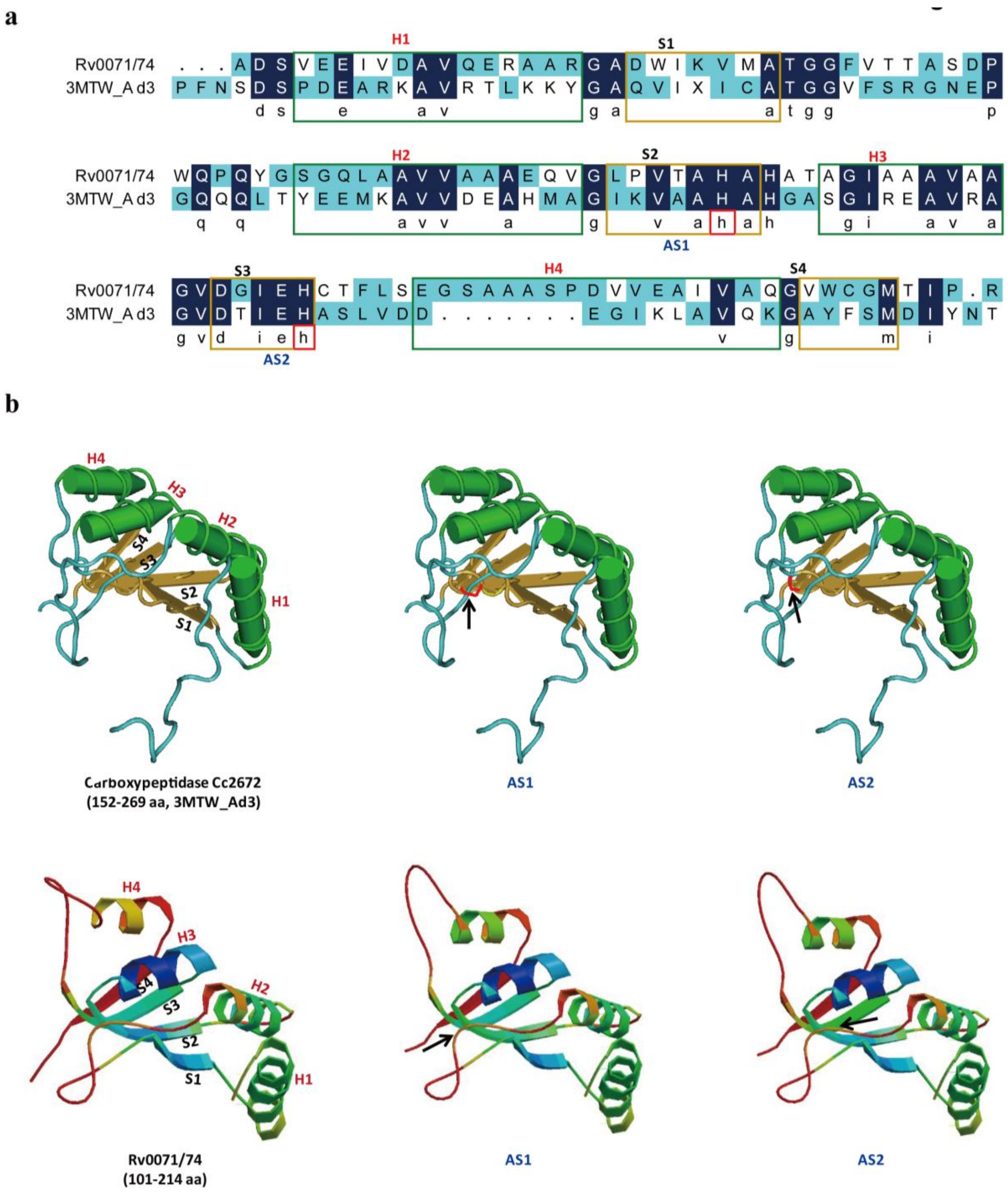

Supplementary Fig. S5 Structure modeling of Rv0071/74 (**a**) Alignment of amino acid sequence between Rv0071/74 (101-214 aa) and carboxypeptidase Cc2672 from *Caulobacter crescentus* Cb15 (PDB ID: 3MTW)_Ad3, 152-269 aa). H: α-helix; S: β-sheet. (**b**) Quaternary structure modeling of Rv0071/74 based on the crystal structure of 3MTW_Ad3 as a template. Point mutation of the two active sites located in β-sheet regions changed the modeled quaternary structure of Rv0071/74. Two active sites of carboxypeptidase Cc2672 are shown by black arrow.

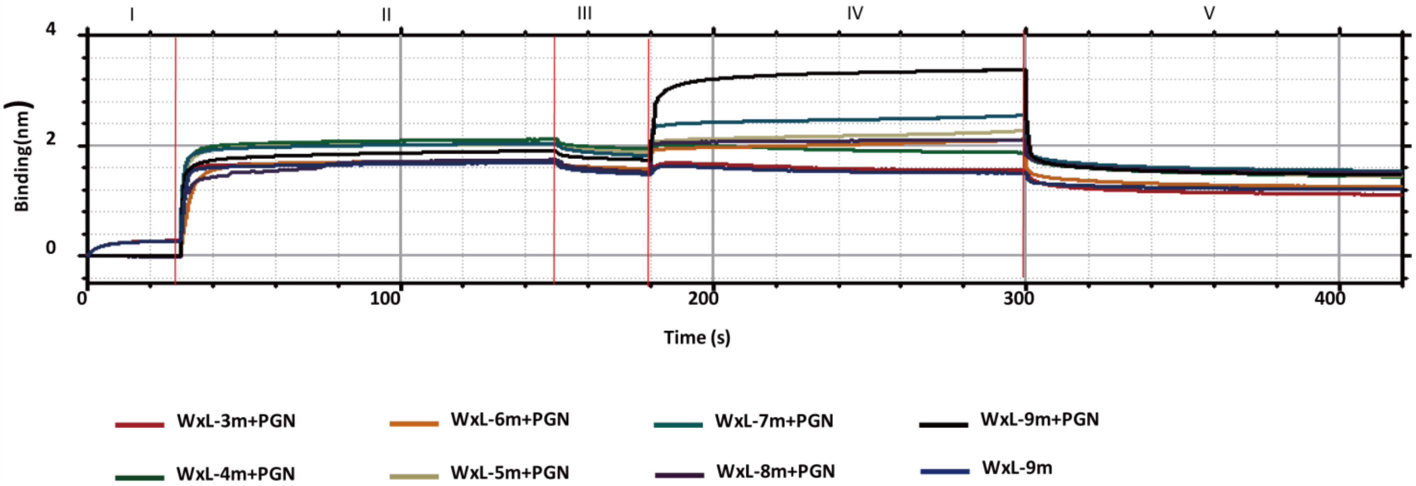

Supplementary Fig. S6 Variable Binding of WxL domain with different alleles with PGN Processed kinetic data for the interaction of WxL domain with peptidoglycan including baseline (phase I), loading the WxL peptide into the sensor (phase II), removing unbound peptide (phase III), association of peptide and peptideglycan (phase IV), and dissociation of peptide and peptideglycan (phase V). Data shown are representative of at least three independent experiments.
