# Supplementary material for: A deletion in the RD105 region confers resistance to multiple drugs in Mycobacterium tuberculosis
Source: BMC Biol. 2019 Jan 25;17:7. doi: 10.1186/s12915-019-0628-6 (PMC6347829; doi:10.1186/s12915-019-0628-6)
Supplement: Supplementary file 2 — Table S1. Final concentrations of antibiotics in drug susceptibility test. Table S3. Drug susceptibility of recombinant M. smegmatis transformed with Rv0071 or Rv0074. Table S4. Drug susceptibility of recombinant M. smegmatis transformed with Rv0071/74-9 m carrying point mutation of AS1 or AS2. (DOCX 23 kb) [file 12915_2019_628_MOESM2_ESM.docx]

**Table S1. Final concentrations of antibiotics in drug susceptibility test**

| **No.** | **Name** | **Abbreviation** | **Final concentration (μg / mL)** | **Cutoff value (≧ μg / mL)** | **Drug type** |
| --- | --- | --- | --- | --- | --- |
| 1 | Streptomycin | SM | 0.25 to 32 | 1 | Aminoglycoside |
| 2 | Amikacin | AMK | 0.25 to 32 | 1 |  |
| 3 | Isoniazid | INH | 0.06 to 8 | 0.25 | Sulfonamide |
| 4 | Ethambutol | EMB | 0.25 to 32 | 1 |  |
| 5 | Clofazimine | CFZM | 0.06 to 8 | 0.25 |  |
| 6 | Rifampin | RIF | 0.25 to 32 | 1 | Rifamycin |
| 7 | Cefoxitin Sodium | CFXS | 0.50 to 64 | 2 | Cephalosporin |
| 8 | Ofloxacin | OFX | 0.125 to 16 | 0.5 | Fluoroquinolone |
| 9 | Linezolid | LZD | 0.06 to 8 | 0.25 | Oxazolidine |

**Table** S3**. Drug susceptibility of recombinant *M. smegmatis* transformed with Rv0071 or Rv0074**

| **Strain** | **MIC （μg/mL）** | | | | | | | | |
| --- | --- | --- | --- | --- | --- | --- | --- | --- | --- |
|  | **SM** | **RFP** | **INH** | **EMB** | **AMK** | **CFZM** | **CFXS** | **OFX** | **LZD** |
| *M. Smegmatis* +pVV16 | 1 | 16 | >8 | ≤0.25 | 1 | 2 | 1 | 0.5 | 0.125 |
| *M. Smegmatis +*pVV16::0071 | 2 | 32 | 8 | 1 | 2 | 4 | 2 | 0.5 | 0.5 |
| *M. Smegmatis +*pVV16::0074 | 4 | >32 | >8 | 1 | 2 | 4 | 4 | 1 | 0.5 |
| *M. Smegmatis +*pVV16::9m | >32 | >32 | >8 | >32 | >32 | >8 | >64 | 4 | 2 |

SM-Streptomycin, RFP- rifampicin, INH- isoniazid, EMB-Ethambutol, AMK-Amikacin, CFZM- Ceftazidime, C

**Table S4. Drug susceptibility of recombinant *M. smegmatis* transformed with Rv0071/74-9m carrying point mutation of AS1 or AS2**

| **Strain** |  | | **MIC （μg/mL）** | | | | | | | |
| --- | --- | --- | --- | --- | --- | --- | --- | --- | --- | --- |
|  | **SM** | **RFP** | | **INH** | **EMB** | **AMK** | **CFZM** | **CFXS** | **OFX** | **LZD** |
| *M. Smegmatis* +pVV16 | 1 | 16 | | >8 | ≤0.25 | 1 | 2 | 1 | 0.5 | 0.125 |
| *M. Smegmatis +*pVV16::9m | >32 | 32 | | >8 | >32 | >32 | >8 | >64 | 4 | 2 |
| *M. Smegmatis +*pVV16::9m-AS1-m | 1 | 8 | | 8 | ≤0.25 | 1 | 4 | 2 | 0.5 | 0.5 |
| *M. Smegmatis +*pVV16::9m-AS2-m | 1 | 8 | | >8 | 0.5 | 1 | 4 | 8 | 0.5 | 0.5 |

SM-Streptomycin, RFP- rifampicin, INH- isoniazid, EMB-Ethambutol, AMK-Amikacin, CFZM- Ceftazidime, CFXS- Cefoxitin Sodium, OFX-Ofloxacin, LZD-Linezolid
